# Assessing pediatric visual acuity with a computerized optokinetic nystagmus analyzer

**DOI:** 10.1016/j.clinsp.2025.100671

**Published:** 2025-04-30

**Authors:** Boyang Zhu, Min Yang, Xiaonan Liu, Bin Yuan, Yuqian Yin, Che Cheng, Faisal UL Rehman

**Affiliations:** a School of Clinical and Basic Medicine, Shandong First Medical University, Shandong, China; b Center for Disease Control and Prevention, Shibei District, Qingdao, Shandong, China; c Department of Ophthalmology, Qingdao Municipal Hospital, Qingdao, Shandong, China; d Department of Management and Marketing, University of Petroleum Huadong, Qingdao, Shandong, China; e Precision Medicine Center of Oncology, The Affiliated Hospital of Qingdao University, Qingdao, Shandong, China; f Department of Research and Development, Pengfengcheng Medical Technology, Qingdao, Shandong, China

**Keywords:** Ophthalmology, Computerized optokinetic nystagmus, Artificial intelligence, Visual acuity, Children

## Abstract

**Objectives:**

To investigate the practicality, accuracy, and consistency of a computer-based Optokinetic Nystagmus (OKN) analyzer for vision diagnosis in preschoolers.

**Design:**

A comparative study evaluating the performance of a computerized OKN analyzer against the traditional E-word visual acuity chart in assessing visual acuity in preschool children.

**Participants:**

A total of 138 children aged 2 to 6 years were enrolled in the study, divided into five age groups: 2 years (29 children), 3 years (27 children), 4 years (28 children), 5 years (28 children), and 6 years (26 children).

**Methods:**

Visual acuity was tested using both the nystagmus meter and the E-word visual acuity chart. Statistical analyses, including correlation coefficient calculation and chi-square tests, were conducted to evaluate the testability and consistency of the methods.

**Results:**

Out of 138 participants, 125 (90.6 %) successfully completed the test using the computerized OKN analyzer. There was no significant difference in the measurability across different age groups. The testability of the E-word visual acuity chart was significantly lower at 71.0 %. A strong correlation (R = 0.872) was found between the results from the nystagmus meter and the E-word visual acuity chart, indicating high consistency between the two methods.

**Conclusion:**

The computerized OKN analyzer is a reliable, accurate, and practical tool for assessing visual acuity in preschoolers. It offers a non-invasive alternative to traditional methods, with high testability and strong correlation with standard visual acuity tests, making it a valuable tool for early detection of vision impairments in young children.

## Highlights

• Computerized optokinetic nystagmus testing improves pediatric vision accuracy.
• Non-invasive OKN analyzer enables early detection of vision impairments in children.
• Innovative OKN technology advances pediatric ophthalmology diagnostic methods.

## Introduction

The detection of early childhood vision impairments relies on accurately measuring visual acuity in young children, which may be a tough task. It is common practice to utilize the same methods used on adults when testing the visual acuity of young children. When testing becomes problematic due to uneasy participants or a language barrier, objective assessments of visual acuity might be beneficial since these subjective approaches depend on the ability and desire of the person to communicate. Also, the current pediatric vision exams have varying degrees of accuracy^1^ With the use of sophisticated algorithms, Artificial Intelligence (AI) in ophthalmology has improved the reliability and efficiency of visual acuity testing, leading to new possibilities for the detection and follow-up of visual abnormalities in children^2^ Automated diagnostic procedures and more accurate clinical assessments are possible when artificial intelligence and machine learning techniques are used to process the nystagmus meter data. Detecting optokinetic nystagmus might be a solution to the difficulty of accurately assessing visual function in this age range using objective techniques.

Optokinetic Nystagmus (OKN) is a rhythmic eye movement that occurs in response to a visual input that is both moving and persistent and even in infants this kind of eye movement can be seen^3^^,^^4^ This eye movement permits the eye to focus and track a distinct input feature. It is characterized by a slow tracking phase and a subsequent resetting motion in the opposite direction^5^ This method requires less assistance from participants because OKN is an involuntary response. By reacting to a carefully designed stimulus, the OKN may identify visually severe impairments that need medical attention^6^^,^^7^ Given the involuntary nature of OKN, it offers a possible way to quickly and correctly test Visual Acuity (VA) in patients who are unable to communicate verbally, particularly younger children^8^ The visual angle subtended by the narrowest stripe width that still causes an eye movement is used in this testing procedure to determine visual acuity^9^ Additionally, Optokinetic Nystagmus (OKN) testing in response to a computer-generated drifting stimulus or a patterned rotating drum may be used to objectively evaluate visual function independent of behavioral reactions^10^ Prior studies have measured optokinetic visual acuity in an OKN apparatus and compared the results with conventional Snellen visual acuity findings in an effort to link subjective and objective visual acuity^11^^,^^12^ Although OKN is often used to determine whether a visual function is present or not, one may objectively quantify resolution acuity by adjusting the spatial frequency of the inducing pattern^13^ Rapid, precise, and controlled clinical evaluation of visual function in this age group may be possible with the use of computer-aided assessment and analysis^14^ Using an automated optokinetic nystagmus analyzer, the authors tap into the potential of AI to streamline and enhance detection procedures, allowing for consistent and dependable evaluation of visual acuity in young children. One non-invasive electronic method for monitoring the eyes is camera-based eye tracking, which usually involves using Infrared (IR) video to extract the ocular signal^15^^,^^16^ The contrast and spatial dimensions of the stimuli may both be precisely adjusted using computer-assisted equipment. The authors considered recognition visual acuity levels as the standard and wondered whether the new technology would allow a more precise clinical evaluation of visual function in preverbal children. The authors selected children between the ages of 2 and 6 for the research group, as it would be difficult to acquire recognition visual acuity measurements using conventional distant visual acuity on children less than 2 years of age. Clinicians may be able to more easily, quickly, and accurately assess visual function in this age group with the use of computer-assisted assessment and analysis. In this study, the authors compared computerized optokinetic nystagmus with the E-word table test. The use of Computerized Optokinetic Nystagmus Analyzer in this case not only increases test accuracy but also advances medical imaging as a whole, as AI-driven solutions are becoming more and more important for early diagnosis and intervention.

## Methods

### Optotype vision screening

**Fig. 1:**
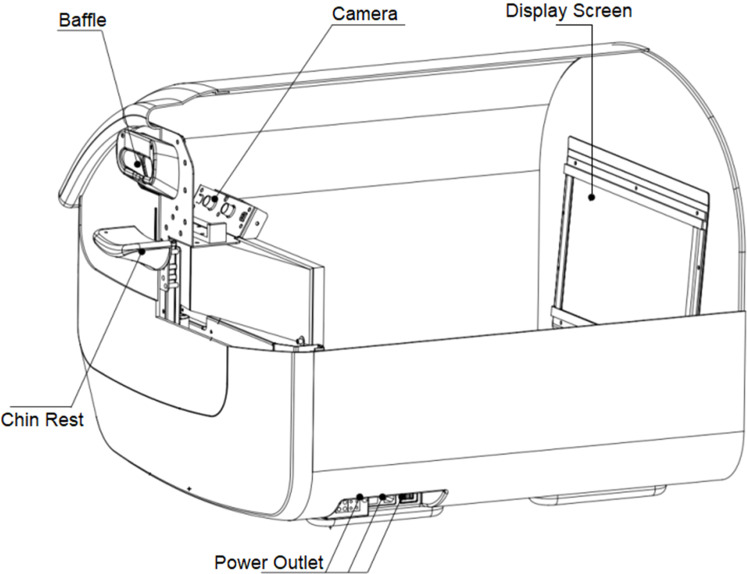
Vision test system.

**Fig. 2:**
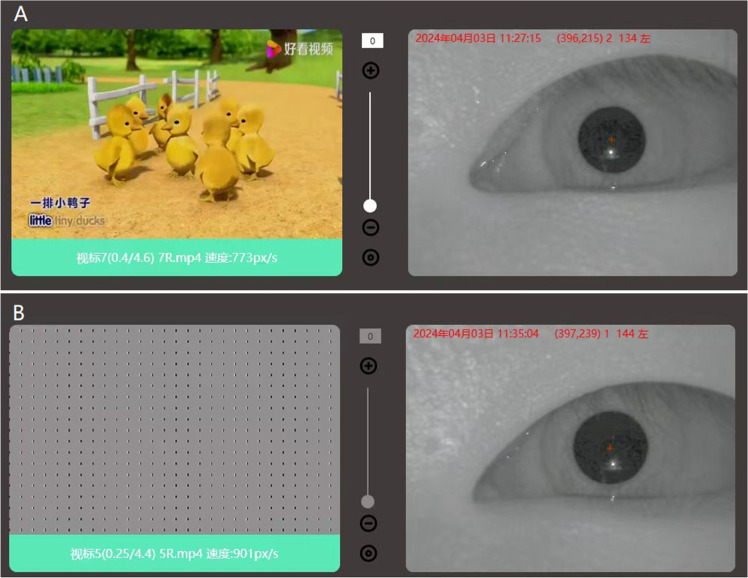
(A) The animations on screen. (B) Scrolling screen bars.

**Fig. 3:**
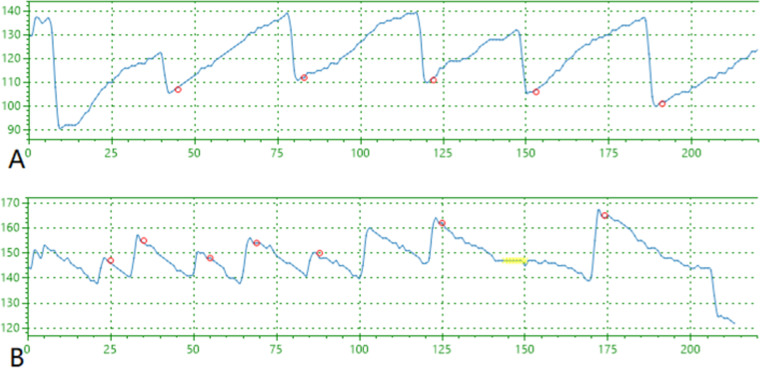
Showing the nystagmus waveform captured by computer where (A) is left eye and (B) is right eye. The vertical axis shows the eye movement, and the horizontal axis is showing the time duration.

Eye vision acuity was recorded with the PFC-001-V1 Vision Test System monocularly the system setup is shown in Fig. 1. The functional structure and working principle of the auxiliary device include an automatic guide rail left and right eye shielding used to shield the eyes during inspection. The chin rest can hold the head still, and the height can be adjusted up and down by pressing the button. The internal display of the machine simulates the alternating rotation when a black and white stripe is scrolled on a 27-inch LED display screen placed 60 cm from the eye with the resolution of the display screen 2560 × 1440. The sight mark is designed to be 0.5‒8.7 mm wide with a distance of 12‒26 mm between grids and with a rolling speed of 5 °/s for the bar grid. The screen played a short video of random animations to relax and make the interest of subjects for the test after the animations the bar grid was played to induce OKN and the values were recorded as shown in Fig. 2. Infrared black and white camera captures dynamic video of human eye nystagmus in real-time. Utilizing infrared LED light, which emits light at 940 nm, the camera can capture clearer images of the eyes. For the duration of the test, a rolling grid scrolls in both directions for a total of 7 seconds: 3.5 seconds from left to right and 3.5 seconds from right to left. The computer will manage the grating speed while maintaining consistent brightness and contrast. When nystagmus is detected, the infrared camera simultaneously sends the information to the computer, which creates a nystagmus waveform as shown in Fig. 3. Every cycle, the patient is considered to have seen the optotype if the rolling grid optotype generates two or more distinctive nystagmus waveforms. If the equipment captures less than two distinct nystagmus waves, it indicates that the individual cannot see the target.

### The order of scrolling optotypes

Optokinetic nystagmus is induced using the induction approach, and the visual aids are presented in a binary order. The size of the visual acuity output by the computer in the first round is equivalent to the visual acuity value on the 0.5 Decimal LogMAR chart. If the regular nystagmus of a subject can be induced and the computer can record at least 2 typical nystagmus waveforms, then the size of the visual acuity output by the computer in the next round is equivalent to the visual acuity value on the 0.3 Decimal LogMAR chart; If the size of the visual acuity output by the computer in the first round is equivalent to the visual acuity value on the 0.5 Decimal LogMAR chart, and cannot induce regular nystagmus in the subjects, or if the computer cannot record at least two typical nystagmus waveforms, then the size of the visual acuity output by the computer in the next round is equivalent to the visual acuity value on the 0.7 Decimal LogMAR chart. Based on this pattern, the authors continued to deduce until we finally found the smallest visual acuity that can trigger nystagmus, which is the visual acuity value of the nystagmus meter.

### The random E-acuity test

**Fig. 4:**
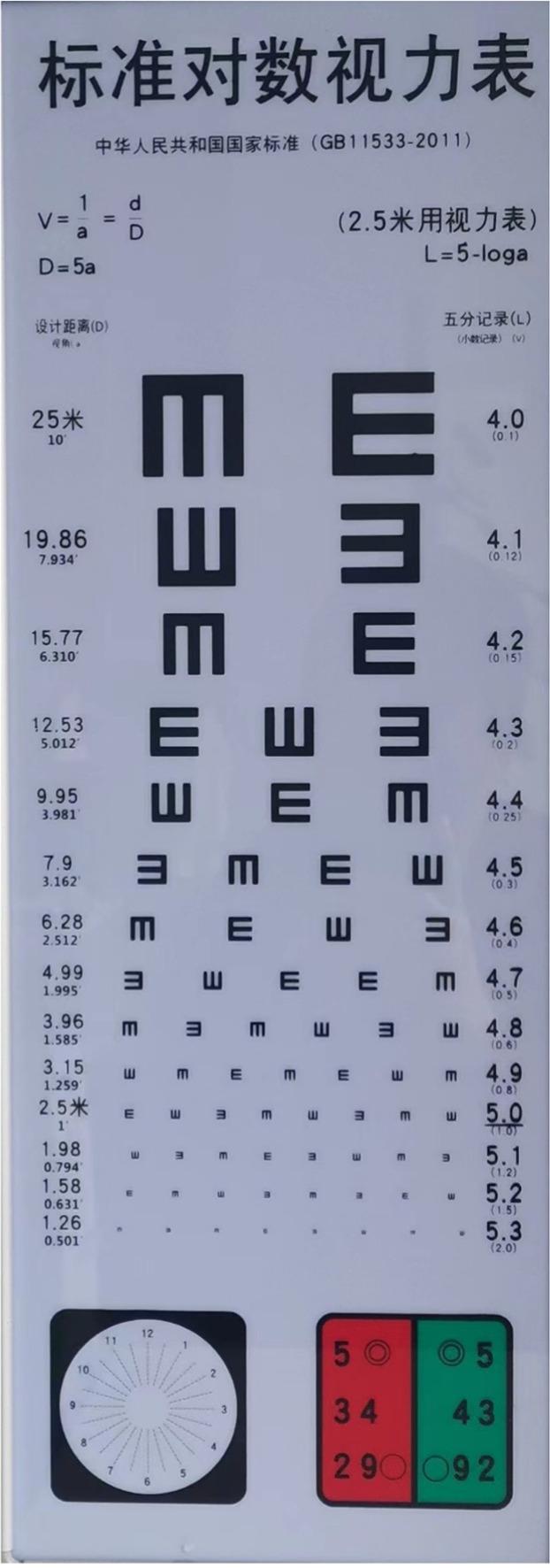
E-word acuity test.

Commercially accessible with the National Standard of the People's Republic of China (GB11533-2011) shown in Fig. 4, this test employs an eye chart known as “E” chart and is intended for those who are unable to read alphabetical letters. The “E” chart differs from the Snellen chart in that it just has a single letter “E” printed in varying sizes and facing in various directions, with no rows of other letters. The letter E is flipped, rotated, and positioned differently as it becomes smaller. The difference between this test and the Snellen is that instead of reading out the letters until they become invisible, one has to determine which direction “E” letters are facing and the test is completed when it is not possible to determine which way the letter is facing.

### Subject data

The test was randomized between nystagmus acuity and “E” acuity chart after removing the spectacles if the subject wore any, a total of 138 ophthalmology subjects aged 2 to 6 years old (5 ± 0.8 years-old) were selected. The subjects were divided into five groups according to their age 2 years (29 cases), 3 years (27 cases), 4 years (28 cases), 5 years (28 cases), and 6 years (26 cases) group. All guardians of the subjects were informed of the research background, research objectives, examination process, and methods. The subjects voluntarily participated in the test, Informed consent was obtained, and the guardian agreed to the test and was informed of the right to withdraw from the test at any time. This study was approved by the Ethics Committee of Qingdao Municipal Hospital (2023 Linshen nº Y009) and conformed to the tenets of the Declaration of Helsinki.

### Method for inspection

E-word visual acuity Test: Every participant consistently checked their uncorrected distance vision from a distance of 5 meters using the E-word visual acuity chart. Reading smaller rows of letters, beginning at the top with the largest row of letters, continued until the orientation of the E letter became unclear, and visual acuity was taken as the recorded visual acuity.

Nystagmus meter Test: Guided the subject to place their lower jaw on the mandibular support and lightly leaned their forehead against the optokinetic nystagmus device. The baffle blocked the view of one eye on the nystagmus meter, while the subject watched the animation on the screen prior to the test, allowing them to fully relax their eyes and establish interest in the test for more compatibility. The infrared camera system recorded the nystagmus and input the recorded nystagmus waveform into the computer. After that, to ascertain if the nystagmus was substantial, the computer filtered out significant waveforms Based on the results of this round of testing, the computer provided the next round of optotypes for the subject to observe until it finally found the smallest optotype that can induce the nystagmus in subject.

### Criteria for inclusion and exclusion

Inclusion criteria included subjects who can pass the E-word visual chart examination in routine outpatient visits. Exclusion criteria included subjects who cannot pass the E-word visual chart examination in routine outpatient visits, and patients suffering from congenital or secondary nystagmus.

### Statistical methods

Used the correlation coefficient method to calculate the correlation between the test results of these two methods and observed if there is a difference in the correlation between the two measurement methods for each age group. The completion rate for every age group was noted, and any discrepancies between age groups were examined. Analyzed the repeatability of the two visual acuity readings obtained from the nystagmus meter and examined whether there were any variations in repeatability across age groups. The *χ*^2^ test was used to calculate the p-value for the comparison between the age groups where p < 0.05 was considered statistically significant. The correlation between the subject's nystagmus meter visual acuity and E-table visual acuity was analyzed using SPSS software 21.

## Results

### Visual acuity rate

Among 138 subjects, 125 subjects passed the test, and the total testability was 90.6 %. The measurability of each age group is shown in Table 1. There is no statistically significant difference in the measurability of each age group (p > 0.05). The total measurability of the E-word table was 71.0 % as shown in Table 2 (p < 0.001).

**Table 1:** Testability results of nystagmus for different ages.

Group	Subjects	Pass the test	Fail the test	Measurability (%)	p-value
2 Years	29	27	2	93.1	p = 0.609
3 Years	27	24	3	88.9	
4 Years	28	23	5	82.1	
5 Years	28	27	1	96.4	
6 Years	26	24	2	92.3	
Total	138	125	13	90.6

**Table 2:** Testability of two visual acuity detection methods.

Method	Subjects	Pass the test	Fail the test	Measurability (%)	p-value
OKN	138	125	13	90.6	p < 0.001
E-word table	138	98	40	71.0	

p < 0.001, indicating that there is an extremely significant statistical difference in measurability between OKN and E-word tables.

### Relationship between E-word visual acuity and nystagmus meter

**Fig. 5:**
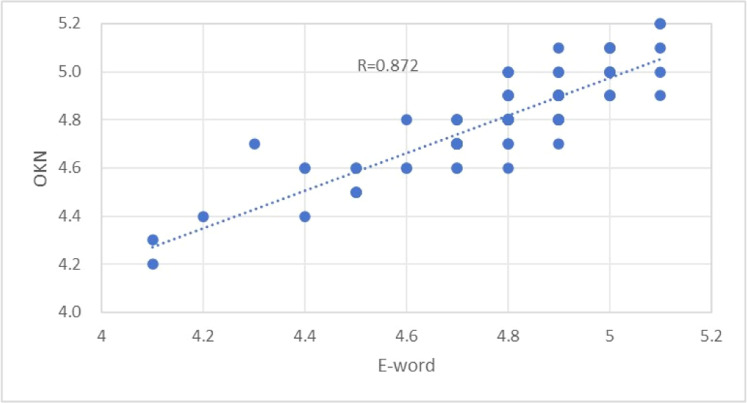
Correlation between the two detection methods for all subjects.

The correlation coefficient between the nystagmus meter test results and the E-word test results of the 89 subjects who completed the nystagmus meter test was R = 0.872, showing a strong correlation, as shown in Fig. 5. The correlations between different groups of the two methods are shown in Table 3. The correlation coefficient R = 0.872, which is greater than 0.8, shows a significant strong correlation. The proportion of visual acuity within 2 lines of the visual acuity chart was 98.8 %. The proportion of visual acuity differing within 1 line from the visual acuity chart was 84.34 %.

**Table 3:** Correlation analysis between OKN vision and E-word table vision.

Group	Number of Samples	Correlation coefficient (R)
3 Years	18	R = 0.813
4 Years	17	R = 0.961
5 Years	24	R = 0.607
6 Years	23	R = 0.886
Total	89	R = 0.872

## Discussion

The reflective and rhythmic eye movements that occur when a moving image is displayed in front of the eyes are known as optokinetic nystagmus. A number of anatomical structures are involved, including the paramedian pontine reticular formation, the occipital lobe, the lateral geniculate body, the macula, and the cerebellar flocculus. The OKN test has been shown to be a viable method for assessing visual function in several studies^17^, ^18^, ^19^ These days, hand-held OKN drums are mostly replaced by computer-assisted OKN testing instruments that provide the ability to alter stripe width and contrast. The computer screen stimulates a bigger visual area, which makes it simpler to focus the youngster on the stimuli in the first place. This is crucial because, particularly in young children, a negative reaction to OKN may be misinterpreted due to a simple lack of attention to the stimuli. The use of artificial intelligence not only streamlines the operational components of the optokinetic nystagmus analyzer but also assures that the analysis of visual responses is nuanced and scalable, providing a strong tool for wider clinical adoption in pediatric ophthalmology. Additionally, useful features of the computer-based system include consistent space-average brightness, uniform rate of movement, and precisely regular, smooth stripes on the screen. The study revealed that 125 of the 138 individuals who completed the screening requirements passed the test, and the testability rate was 90.6 %, demonstrating the good operability of this method. The consistent brightness of the nystagmus equipment, the stable optotype movement speed throughout the test, and the lack of distraction from the surroundings of the examination room might be the reason for the good repeatability of the vision assessed by the nystagmus meter. The disadvantage of the wheel effect brought on by the tympanic method is minimized in this research by varying the width between the gratings, and the speed of grating is designed to steadily produce nystagmus in the participants. The combination of AI and medical imaging in the study framework demonstrates the potential for these technologies to revolutionize not only the analysis but also the interpretation of complicated diagnostic data, opening the path for advances in non-invasive ophthalmologic investigations.

Future developments in pediatric ophthalmology might involve the use of artificial intelligence to transform early diagnostic procedures, especially for disorders like astigmatism and amblyopia, where a timely diagnosis can have a major impact on patient outcomes and treatment plans^20^ The visual acuity of the nystagmus meter and the outcomes of the E-word visual acuity chart were compared in this study. The results showed that there was a significant negative association between the visual acuity measured by the nystagmus instrument and the visual acuity determined by the other acuity chart techniques, which was in line with previous studies^21^^,^^22^ Using the optokinetic nystagmus approach, test results may be compared with the standard worldwide visual acuity chart to provide a useful comparison relationship. The use of artificial intelligence in the computerized optokinetic nystagmus analyzer is a big step forward in ophthalmology, providing a unique tool for objectively assessing children's visual acuity with excellent reliability. In the current study, there is a correlation between the visual acuity value determined by the E-word visual acuity chart and the visual acuity value acquired by the nystagmus meter visual acuity test. A number of factors contributed to the failure of the nystagmus test, including tension esotropia, crying throughout the test, and being unable to finish it due to vertigo. Age factors have no effect on this method, as shown by the lack of a statistically significant difference in the measurability of the age groups.

## Conclusion

The study on visual acuity of preschoolers using the computerized optokinetic nystagmus analyzer provides a practical and efficient tool for early detection and management of vision impairments, enhancing the transition from basic research to clinical interventions and improving visual health outcomes in young children. Results from comparing and analyzing data on measured visual acuity show that the nystagmus meter and the E-word eye chart have a good association. The nystagmus meter can be used as an alternate method of evaluating visual acuity since, the test administered to preschoolers showed good operability, accuracy, and stability.

## Authors’ contributions

Boyang Zhu collected the data, conducted visual acuity tests on subjects, data analysis, and prepared the manuscript. Min Yang, Xiaonan Liu, Bin Yuan and Yuqian Yin data collection and visual acuity tests on subjects. Che Cheng conducted the statistical analysis and assisted in manuscript writing, Faisal UL Rehman data analysis, supervised the research, data analysis and final manuscript editing and writing.

## Declaration of competing interest

The authors declare no conflicts of interest.

## References

[1] Anstice NS, Thompson B. (2014). The measurement of visual acuity in children: an evidence-based update. Clin Exp Optom.

[2] Du X-L, Li W-B, Hu B-J. (2018). Application of artificial intelligence in ophthalmology. Int J Ophthalmol.

[3] Doustkouhi SM, Turnbull PR, Dakin SC. (2020). The effect of simulated visual field loss on optokinetic nystagmus. Transl Vis Sci Technol.

[4] Garbutt S, Harris CM. (2000). Abnormal vertical optokinetic nystagmus in infants and children. Br J Ophthalmol.

[5] Kanari K, Kaneko H. (2019). Effect of visual attention and horizontal vergence in three-dimensional space on occurrence of optokinetic nystagmus. J Eye Mov Res.

[6] Aleci C, Scaparrotti M, Fulgori S, Canavese L (2018). A novel and cheap method to correlate subjective and objective visual acuity by using the optokinetic response. Int Ophthalmol.

[7] Chang LY-L, Guo P, Thompson B, Sangi M, Turuwhenua J. (2018). assessing visual acuity–test-retest repeatability and level of agreement between the electronic ETDRS chart (E-ETDRS), optokinetic nystagmus (OKN), and sweep VEP. Invest Ophthalmol Vis Sci.

[8] Anstice NS, Thompson B. (2014). The measurement of visual acuity in children: an evidence-based update. Clin Exp Optom.

[9] Von Noorden GK. (1996). Theory and management of strabismus.

[10] Hyon JY, Yeo HE, Seo J-M, Lee IB, Lee JH, Hwang J-M. (2010). Objective measurement of distance visual acuity determined by computerized optokinetic nystagmus test. Invest Ophthalmol Vis Sci.

[11] Catford GV, Oliver A. (1973). Development of visual acuity. Arch Dis Child.

[12] Gruber H. (1984). Decrease of visual acuity in patients with clear media and normal fundi objective screening methods for differentiation and documentation. Doc Ophthalmol.

[13] Gorman JJ, Cogan DG, Gellis SS. (1957). An apparatus for grading the visual acuity of infants on the basis of opticokinetic nystagmus. Pediatrics.

[14] Hathibelagal A. (2013).

[15] Al-Rahayfeh A, Faezipour M. (2013). Eye tracking and head movement detection: a state-of-art survey. IEEE J Transl Eng Health Med.

[16] Valenti R, Staiano J, Sebe N, Gevers T. (2009). Image Analysis and Processing–ICIAP 2009: 15th International Conference Vietri sul Mare, Italy, September 8-11, 2009 Proceedings 15.

[17] Shin YJ, Park KH, Hwang J-M, Wee WR, Lee JH, Lee IB. (2006). Objective measurement of visual acuity by optokinetic response determination in patients with ocular diseases. Am J Ophthalmol.

[18] Wester ST, Rizzo 3rd JF, Balkwill MD, Wall 3rd C. (2007). Optokinetic nystagmus as a measure of visual function in severely visually impaired patients. Invest Ophthalmol Vis Sci.

[19] Schwob N, Palmowski-Wolfe A. (2019). Objective measurement of visual acuity by optokinetic nystagmus suppression in children and adult patients. J AAPOS.

[20] Rampat R, Deshmukh R, Chen X, Ting DSW, Said DG, Dua HS (2021). Artificial intelligence in cornea, refractive surgery, and cataract: basic principles, clinical applications, and future directions. Asia Pac J Ophthalmol.

[21] Schwob N, Palmowski-Wolfe A. (2020). Establishing an objective measurement of visual acuity with a computerised optokinetic nystagmus suppression test. Klin Monbl Augenheilkd.

[22] Turuwhenua J, LinTun Z, Norouzifard M, Edmonds M, Findlay R, Black J (2023). OKN-Fast: objective visual acuity threshold measurement using the optokinetic response. Annu Int Conf IEEE Eng Med Biol Soc.

